# New clinical guidelines on the spinal stabilisation of adult trauma patients – consensus and evidence based

**DOI:** 10.1186/s13049-019-0655-x

**Published:** 2019-08-19

**Authors:** Christian Maschmann, Elisabeth Jeppesen, Monika Afzali Rubin, Charlotte Barfod

**Affiliations:** 1 Emergency Department, Copenhagen University Hospital Bispebjerg, Copenhagen, Denmark; 2 Department of Anesthesiology, Copenhagen University Hospital Bispebjerg, Copenhagen, Denmark; 3 Emergency Medical Services Copenhagen, University Copenhagen, Copenhagen, Denmark; 4 Norwegian Trauma Registry, Division of Orthopaedic Surgery, Oslo University Hospital, Oslo, Norway; 5 Faculty of Health Science, University of Stavanger, Stavanger, Norway; 6 Cochrane Anesthesia & Cochrane Critical and Emergency Care Group, Copenhagen, Denmark; 7 Department of Anesthesiology, Copenhagen University Hospital Herlev, Copenhagen, Denmark

**Keywords:** Guideline, Immobilisation, Spinal stabilisation, Trauma, Traumatic spinal cord injury, Rigid cervical collar, Spineboard, Vacuum mattress, Blunt trauma, Penetrating trauma

## Abstract

Traumatic spinal cord injury is a relatively rare injury in Denmark but may result in serious neurological consequences. For decades, prehospital spinal stabilisation with a rigid cervical collar and a hard backboard has been considered to be the most appropriate procedure to prevent secondary spinal cord injuries during patient transportation. However, the procedure has been questioned in recent years, due to the lack of high-quality studies supporting its efficacy. A national interdisciplinary task force was therefore established to provide updated clinical guidelines on prehospital procedures for spinal stabilisation of adult trauma patients in Denmark. The guidelines are based on a systematic review of the literature and grading of the evidence, in addition to a standardised consensus process.

This process yielded five main recommendations:

A strong recommendation against spinal stabilisation of patients with isolated penetrating trauma; a weak recommendation against the prehospital use of a rigid cervical collar and a hard backboard for ABCDE-stable patients; and a weak recommendation for the use of a vacuum mattress for patient transportation. Finally, our group recommends the use of our clinical algorithm to ensure good clinical practice.

**Electronic supplementary material:**

The online version of this article (10.1186/s13049-019-0655-x) contains supplementary material, which is available to authorized users.

## Background

Traumatic spinal cord injury (TSCI) is a relatively rare injury. The overall annual incidence in Denmark during 1990–94 to 2010–12 was 10.2 per million person-years at risk and varied from 8.3 to 11.8 [1]. However, despite its rarity, the consequences of TSCI are serious, and may lead to a substantial handicap.

In order to prevent secondary spinal cord injuries under transportation and medical treatment of trauma patients, it was postulated in the mid-1960s, that this risk might be reduced by stabilisation of the patient using a rigid cervical collar and a hard backboard. This strategy was adopted by many prehospital medical services worldwide as well as on trauma courses such as Prehospital Trauma Life Support (PHTLS®) and Advanced Trauma Life Support (ATLS®) [2, 3]. This change occurred despite a lack of high-quality study data to suggest clear benefits [4–6]. On the contrary, a growing body of evidence during recent years indicates that the use of the rigid cervical collar and the hard backboard might indeed have harmful effects. The two most important studies are presented below.

A study published by Hauswald et al. in 1998 described a 5-year retrospective chart review at two university hospitals (University of Malaya, Malaysia and University of New Mexico, USA) where the effect of emergency spinal stabilisation was examined in relation to neurological outcome for patients with blunt traumatic spinal injuries [7]. All patients with acute blunt TSCI who were transported directly from the injury site to the hospital were included. The two hospitals were comparable with respect to physician training and clinical resources. None of the 120 patients examined at the University of Malaya underwent spinal stabilisation during patient transportation, whereas all 334 patients examined at the University of New Mexico did.

The study found that there were fewer neurologic disabilities sustained in the Malaysian patients who did not undergo spinal stabilisation and concluded that there was less than a 2% chance that spinal stabilisation had any beneficial effect on neurologic outcomes in patients with blunt TSCI.

In 2010, Haut et al. published a study based on a retrospective analysis of penetrating trauma patients in the US American National Trauma Data Bank [8]. They studied more than 45,000 cases and their results showed that only 30 (0.01%) had incomplete spinal cord injury and underwent surgical spinal fixation. The number needed to treat (NNT) with spinal stabilisation to potentially benefit one patient was 1032. Conversely, the number needed to harm (NNH) with spinal stabilisation to potentially contribute to one death was 66. The authors concluded that prehospital spinal stabilisation was associated with a higher mortality risk in patients with penetrating trauma and therefore should not be routinely used in patients with penetrating trauma.

Numerous studies were published in recent years which reveal further possible hazardous effects of spinal stabilisation, including pain [6, 9–12], the development of pressure ulcers [9, 11–13], elevated intracranial pressure [11], prolonged intrahospital length of stay [14], an increased number of radiological examinations [15–17], an increased difficulty of clinical examination [6], prolonged prehospital on-scene time [11, 12], difficulty in intubation [18] and a risk of spinal fracture displacement in elderly patients [19]. The strength of the evidence in the aforementioned studies was either low or very low according to the GRADE-tool (Grading of Recommendations Assessment, Development and Evaluation) [20].

Based on this growing body of evidence, we have recently published new national guidelines for the spinal stabilisation of adult trauma patients in Danish language through the Danish National Board of Health [21], which are presented here in English language to allow a broader international audience access to our guidelines.

## Methods

A systematic review of the literature was performed involving grading of the strength of the evidence, clinical judgment and a consensus process. In order to involve all relevant stakeholders, an interdisciplinary working group was established consisting of representatives from eight different Medical Associations in Denmark, representatives from the Danish ATLS, PHTLS and International Trauma Life Support (ITLS) chapters, medical directors from the four largest Danish ambulance providers as well as representatives from all five Danish Emergency Medical Services (EMS) (Table 1). The working group also included two research methodologists contributing in the systematic evidence work (EJ and MAR).

**Table 1:** Members of the Danish interdisciplinary working group

Members of the Danish interdisciplinary working group	
• Danish Society for Emergency Medicine – DASEM (chairman) • Danish Neurosurgical Society – DNKS • Danish Society for Spinal Surgery – DRKS • Danish Orthopaedic Society - DOS • Danish Orthopaedic Trauma Society – DOTS • Danish Society for Anesthesiology and Intensive Care Medicine – DASAIM • Danish Society for Radiology - DRS • Danish Society for Ambulance- and Paramedicine – DSAP • ATLS® Denmark • PHTLS® Denmark • ITLS® Denmark • Greater Copenhagen Fire Department – HBR (ambulance services) • Falck A/S (ambulance services) • Responce A/S (ambulance services) • Ambulance Southern Denmark (ambulance services) • EMS Copenhagen • EMS Region North Denmark • EMS Region Central Denmark • EMS Region Southern Denmark • EMS Region Sealand	

The scope of the guideline was defined based on five clinical key questions relating to the population, intervention, comparator/control and outcomes (PICO) (Table 2).

**Table 2:** The PICO questions

Clinical question	Population	Intervention	Comparator	Outcome
Should adult trauma patients where there is concern for the development of a secondary spinal cord injury undergo spinal stabilisation...	Adult trauma patients (> = 18 years), where there is concern for the development of a secondary spinal chord injury			
1.) ...with a rigid cervical collar?	ditto	Rigid cervical collar	No rigid cervical collar	Mortality Neurologic morbidity Ulcerations Pain / discomfort Respiratory deterioration Time to diagnose Intracranial pressure
2.) …on a hard backboard?	ditto	Hard backboard	No hard backboard	Mortality Neurologic morbidity Pain/discomfort Ulcerations Time to diagnose
3.) …in a vacuum mattress?	ditto	Vacuum mattress	No vacuum mattress	Mortality Neurologic morbidity Pain/discomfort Ulcerations Time to diagnose
4.) Should adult trauma patients with isolated penetrating injuries undergo spinal stabilisation?	ditto	Spinal stabilisation	No spinal stabilisation	Mortality Neurologic morbidity
5.) Should the decision, whether and how to stabilise the spine of a trauma patient be facilitated by a clinical decision tool?	ditto	Use of a clinical decision tool	No use of a clinical decision tool	Mortality Neurologic morbidity

We defined the target population as trauma patients aged 18 years or above, who experienced spinal trauma within 48 h, and were at risk of developing a spinal cord injury. This definition was based on practical constraints rather than research evidence.

The initial searches of existing guidelines were performed on October 19th, 2017, and included the following resources: Guidelines International Network (G-I-N), National Institute for Health and Care Excellence (NICE, UK), National Guideline Clearinghouse, Scottish Intercollegiate Guidelines Network (SIGN), UK National Institute for Health Research’s Health Technology Assessment database (NIHR-HTA), Swedish Agency for Health Technology Assessment and Assessment of Social Services (SBU), Swedish National Board of Health and Welfare (Socialstyrelsen), Norwegian Directorate of Health (Helsedirektoratet), Norwegian Institute of Public Health (Kunnskapssenteret), and the Australian Physiotherapy Evidence Database (PEDRO). A Norwegian guideline of best practice which covered literature from 1966 to 2015 was also identified and included in the scoping search [22]. The search strategy from this guideline was extended to include articles from January 2015 to October 2017. The search strategy is described in full in the Additional file 1. A research librarian conducted the systematic search for systematic reviews and primary studies in the databases of MEDLINE, EMBASE, CINAHL and the Cochrane Library. We searched for a combination of subject terms and text words to identify studies relating to spinal cord injuries and spinal stabilisation / immobilisation.

Searches were limited to human studies published in English, Swedish, Norwegian, Danish or German language. Two reviewers independently screened titles and abstracts of all articles identified in the searches for inclusion (EJ and MAR). Any discrepancy was resolved through discussion and consensus in our interdisciplinary working group. We read the full-texts and critically reviewed and included them, if relevant according to the PICO questions. For completeness, we identified additional articles by scanning the reference lists of the included studies and the authors’ contributing papers known to them. We used the Critical Appraisal Skills Program (CASP) checklist for critical appraisal of observational studies and the Appraisal of Guidelines for Research and Evaluation (AGREE II) tool for included guidelines [23, 24]. The critical appraisal of all studies was done by EJ in cooperation with the working group.

As no randomized controlled studies or large observational studies were identified, we systematically reviewed all relevant published material, regardless of the study design. Case reports and cadaver studies were excluded due to the high risk of bias in case studies often involving only one patient and the low generalisability relating to cadaver studies..

The strength of the evidence and strength of recommendations were assessed using the GRADE (Grading of Recommendation, Assessment, Development and Evaluation) approach [20]. The strength of the evidence was rated as high, moderate, low or very low. When assessing the strength of recommendations, we considered two factors and integrated them in a working group consensus process: benefit versus harm and quality of the evidence. The strength of the recommendations was graded as strong or weak or as good clinical practice. Our group evaluated the final national clinical guidelines (from June 2018) in a Delphi/consensus process utilising the Appraisal of Guidelines for Research and Evaluation (AGREE II) tool / Danish National Board of Health’s handbook of methods [25, 26]. A preliminary version of the new guidelines was sent out to all relevant medical associations and the other respective institutions via their representatives from our working group. The chairman of our group or the groups’ representatives answered all comments and questions during two public hearing processes. After each public hearing process our group re-discussed and adapted the guidelines accordingly.

### Implementation and meetings

These guidelines were implemented in Denmark by 1st March 2019. They were first published on 9th October 2018 on the public internet site of the Danish National Board of Health [21] and were shortly after available through several Danish Medical Societies’ homepages. The guidelines were also available publicly via an in-depth Danish podcast [27]. In order to facilitate both pre- and in-hospital implementation, the five Danish Regions funded the production of several open-source videos. These videos were published in December 2018 and are publicly available [28]. Since January 2019, the guidelines form part of the education, certification and recertification of all pre-hospital personnel, both via internal education and through incorporation in the Danish chapters’ PHTLS®-, ATLS®- and ITLS®-courses.

In April 2019, we published a short “heads-up” notice about our guidelines in “Der Notarzt”, a German language medical paper [29].

The guidelines have been presented in Denmark, at the 8th Danish Emergency Medical Conference (DEMC8) 2018, the Annual meeting of the Danish Society of Anesthesiology and Intensive Care Medicine (DASAIM) 2018, the Copenhagen Critical Symposium 2019 [30], and the European EMS Congress 2019 in Madrid, Spain [31].

## Results

**Fig. 1:**
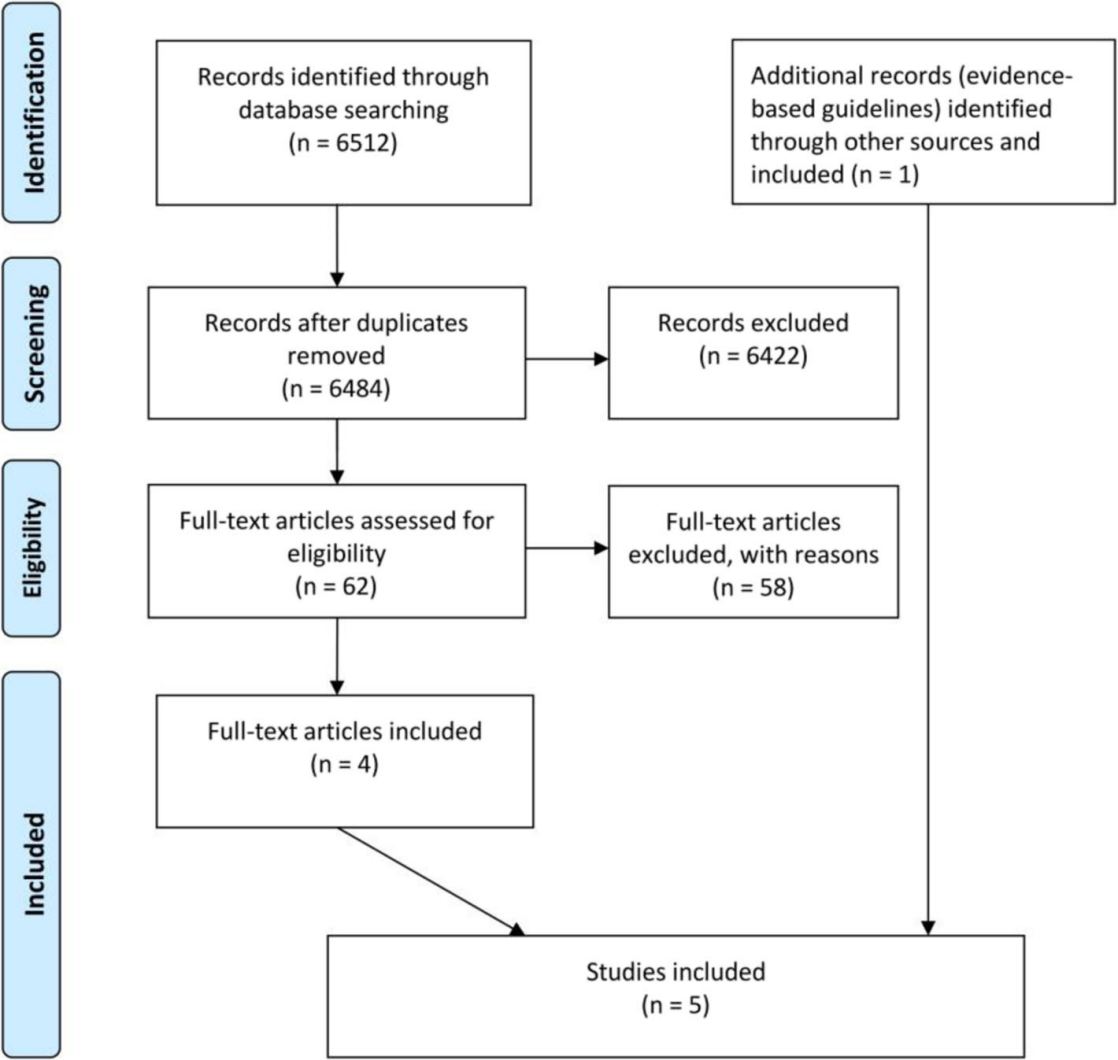
Prisma flow-chart depicting the literature search and selection of included and excluded studies

A total of 6484 titles and abstracts were identified in the systematic review. Of these, four observational studies of moderate and high methodological quality were included, in addition to the included Norwegian guideline with included references. The search process is shown in Fig. 1.

Following main recommendations for spinal stabilisation of adult trauma patients were given based on the identified studies, clinical judgment and consensus decisions made in the interdisciplinary working group (Table 3).

1. Clinical question 1

**Table 3:** Summary of main recommendations, quality of evidence and strength of recommendation

Recommendation	Quality of evidence	Strength of recommendation
Adult trauma patients should not undergo spinal stabilisation with a rigid cervical collar	very low	weak
Adult trauma patients should not undergo spinal stabilisation on a hard backboard unless in case of time-critical ABCDE-unstable patients, where other spinal stabilisation measures would be more time consuming	very low	weak
Adult ABCDE-stable patients with neurologic deficit and / or osseous spinal pain on examination should undergo spinal stabilisation in a vacuum mattress	very low	weak
Adult trauma patients with isolated penetrating injury should not undergo spinal stabilisation	moderate	strong
Our triaging tool should be used in order to facilitate decision on spinal stabilisation	none	good clinical practice

Should adult trauma patients with risk of a secondary spinal cord injury undergo spinal stabilisation with a rigid cervical collar?

### Recommendation

There is a weak recommendation against the use of a rigid cervical collar as a spinal stabilisation measure in adult trauma patients.

### Level of evidence

Very low

### Evidence and rationale

No published high-quality studies were found. The published studies were of very low evidence according to GRADE, mostly due to the fact, that the data was extrapolated from either cadaver studies or studies with healthy volunteers [4]. We did not find any study proving the efficacy of rigid cervical collars with regards to a better neurological outcome or mortality [4, 6, 10, 22, 32–38]. However, several publications describe the efficacy of a rigid cervical collar with regards to the reduction of range of motion in the cervical spine, and all note that the effect on the range of motion in the neck is very limited [32, 37, 39–41].

In 2013, a joint committee from The American Association of Neurological Surgeons (AANS) and the Congress of Neurological Surgeons published new guidelines for the management of acute cervical spine and spinal cord injuries [42]. These guidelines still recommend the use of a rigid cervical collar for the spinal stabilisation of the cervical spine. However, the authors concluded that this recommendation is based on anatomical and mechanical considerations rather than on evidence. Furthermore, they found that the variety of techniques used and the lack of evidence to advocate a uniform device for spinal stabilisation made spinal stabilisation technique and device recommendations difficult.

In line with previous publications, additional publications proposing possible harmful effects were found. For example, longer stay in the emergency room [43], decreased lung function [44], development of pressure ulcers [13, 45], impeded airway management [22], worsening of existing cervical injury [22], severe neurological deterioration in patients with ankylosing spondylitis [22], triggering of non-compliance or agitation and even increased spinal movement due to pain or discomfort [22] as well as possible elevation of intracranial pressure [46].

The weak recommendation is given due to a low prevalence of secondary TSCI as well as the limited efficacy of the rigid cervical collar regarding to the movement in the cervical spine and other existing methods of spinal stabilisation. Moreover, there was a lack of studies demonstrating a positive effect on both survival and neurological outcomes and the increasing evidence for possible harmful side effects when applying a rigid cervical collar. Instead of using the rigid cervical collar for spinal stabilisation where indicated, we recommend using manual in-line stabilisation of the head (the MILS-maneuver), head blocks, or a or a vacuum mattress reaching up over the head [22, 47, 48].

According to GRADE it is not possible to give a stronger recommendation against the use of rigid cervical collars due to the lack of high-quality studies regarding their use.

2.) Clinical question 2

Should adult trauma patients with risk of a secondary spinal cord injury undergo spinal stabilisation on a hard backboard?

### Recommendation

There is a weak recommendation against the use of a hard backboard as a spinal stabilisation measure in case of ABCDE-stable patients.

### Level of evidence

Very low

3.) Clinical question 3

Should adult trauma patients with risk of a secondary spinal cord injury undergo spinal stabilisation on a vacuum mattress?

### Recommendation

There is a weak recommendation for the use of a vacuum mattress as a spinal stabilisation measure for ABCDE-stable patients with neurologic deficit and / or osseous pain on examination.

### Level of evidence

Very low

Evidence and rationale for clinical question 2 and 3:

Our group did not find any published high-quality studies covering the efficacy of a rigid backboard for spinal stabilisation. The strength of evidence in all published studies was very low according to GRADE, mostly due to the fact that the data was extrapolated from either cadaver studies or studies with healthy volunteers [4]. Besides the lack of studies supporting improved patient outcome when transporting trauma patients on a hard backboard, there are two studies highlighting the previous mentioned adverse effects. This includes the possible development of significant discomfort and moderate to severe pain after a short time on the hard backboard, possible voluntary spinal movement and even the possible development of pressure ulcers after prolonged exposure [6, 22]. Furthermore, the efficacy of the hard backboard with regards to restriction of lateral movement under ambulance transport compared to a simple ambulance stretcher is also questionable [49].

Several studies favor the use of soft surface stretcher systems, e.g. the vacuum mattress. This in order to reduce the above mentioned possible adverse effects of the hard surface stretcher systems and at the same time maintain the principle of a minimal handling strategy [18, 22, 50–52]. Moreover, some studies suggest that the vacuum mattress may provide either a similar or even superior degree of spinal stabilisation compared to the hard backboard [22, 48]. Because of this, we recommend the use of a vacuum mattress over the use of a hard backboard for patient transportation of adult trauma patients undergoing spinal stabilisation.

4.) Clinical question 4:

Should patients with isolated penetrating injuries undergo spinal stabilisation?

### Recommendation

There is a strong recommendation against the effort of a spinal stabilization in patients with isolated penetrating injuries.

### Level of Evidence

Moderate

### Evidence and rationale

Patients with penetrating injuries may be ABCDE-unstable and in need of time-critical surgical intervention.

In 2010, Haut et al. published a retrospective analysis of the National Trauma Data Bank, studying 45,284 patients with isolated penetrating trauma [8]. They compared outcomes between patients who received spinal stabilisation and patients who did not. The results showed that unadjusted mortality was twice as high in the patients who underwent spinal stabilisation (14.7% vs. 7.2%, *p* < 0.001) compared to the patients that did not. The odds ratio of death for patients undergoing spinal stabilisation was 2.06 (95% CI: 1.35–3.13) compared to the patients that did not, probably due to the prolonged prehospital time used in the spinal stabilisation of the patients. Out of the 45,284 patients only 30 (0.01%) patients had incomplete spinal cord injury and underwent subsequent spinal surgery. The NNT with spinal stabilisation to potentially benefit one patient was 1032, whereas the NNH was 66 [8].

Due to the study’s effect size and the high number of patients studied, the study was upgraded to a moderate level of evidence according to GRADE.

5.) Clinical question 5:

Should the decision, whether and how to perform spinal stabilisation on an adult trauma patient be facilitated by a clinical triaging tool?

### Recommendation

It is good clinical practice to use our clinical triaging tool to determine whether and how to perform spinal stabilisation on an adult trauma patient.

### Level of evidence

None. Good clinical practice

### Evidence and rationale

**Fig. 2:**
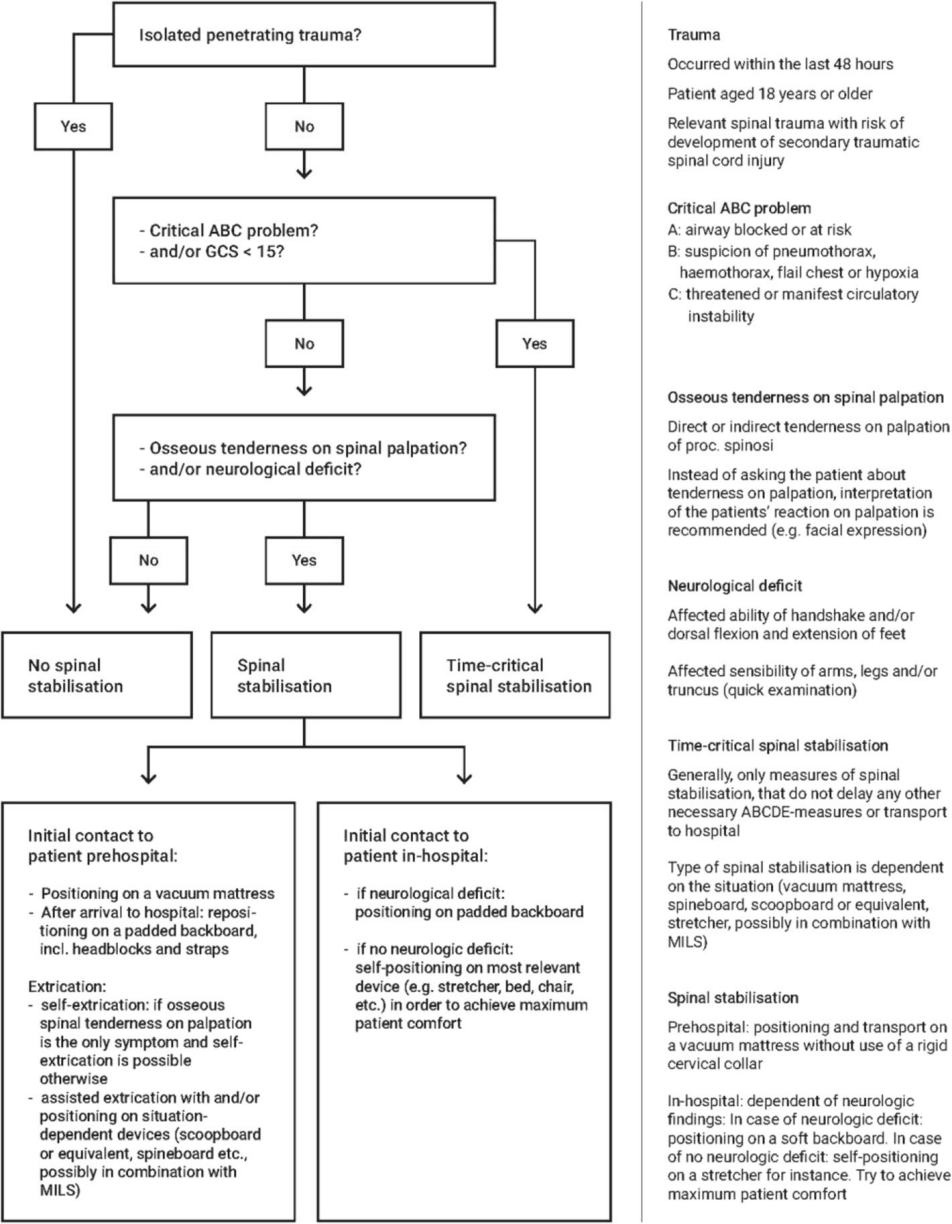
Algorithm for a clinical handling strategy with spinal trauma

It is very unlikely that all patients with a spinal injury need spinal stabilisation in order to prevent them from developing a secondary spinal injury. But how can we determine, which patients need spinal stabilisation and which do not? Studies have shown that prehospital triaging tools based on mechanisms of injury instead of clinical findings are inferior with regards to accuracy and lead to over-triage [53–55]. Several EMS systems around the world are already using different triaging tools facilitating the decision whether to perform spinal stabilisation [22, 56, 57]. Most of these triaging tools are traditionally based on decision aids like the National Emergency X-radiography Utilisation Study (NEXUS) tool or the Canadian C-Spine Rule criteria (CCR). Originally, these decision aids were developed to help clinicians to decide whether a patient needs radiographic imaging in order to diagnose spinal injuries [58, 59]. In order to further reduce over-triage, our group modified these earlier published triaging tools and developed a new clinical decision tool illustrated in Fig. 2.

We recommend assigning adult trauma patients to one out of three groups:

1. No efforts of spinal stabilisation
2. Spinal stabilisation on a vacuum mattress
3. Time-critical spinal stabilisation

In line with earlier publications we agree that alert and ABCDE-stable patients will seek to stabilise their spine themselves and in the most comfortable position for them as possible automatically [35, 60].

We do also recommend that patients being affected by alcohol or drugs should be treated like all other non-intoxicated patients, since it is clinically difficult to differentiate between clinical findings resulting from intoxication or from other more critical injuries such as intracranial hemorrhage [61].

Furthermore, we recommend that patients with so-called “distracting injuries” and a GCS of 15 should be treated like all other alert patients. It has been seen that a so-called distracting injury does not affect the sensitivity of an examination of the cervical spine [62–64].

When it comes to the clinical examination of the spine, we recommend using the interpretation of the patient’s face expression as a marker of pain, rather than asking the patient directly. Our concern was overtriage by overestimation of symptoms through the use of leading questions [65, 66].

In general, our group still supports a minimal handling strategy, but acknowledges potentially life-threatening injuries that might demand immediate intervention. In these instances, we therefore recommend a so-called time-critical spinal stabilisation, which must not delay other life-saving procedures or transportation. Our group cannot recommend a standard procedure of a time-critical spinal stabilisation, since it should be based on the individual patient’s situation and other factors, such as the availability of stabilisation- and transportation tools. Therefore, a time-critical spinal stabilisation might consist of the use of a vacuum mattress, a hard backboard, a scoop stretcher or a simple ambulance stretcher, as well as the MILS maneuver; depending on the most appropriate solution for the given situation. With respect to the transportation of unconscious, non-intubated trauma patients, our group supports the use of the novel lateral trauma position (LTP) or other positioning maneuvers like the HAINES-maneuver (high-arm-IN-endangered-spine) for time-critical spinal stabilisation. This is in line with previous studies, which suggest that these maneuvers do not produce more movement in the unstable spine than the traditional log-rolling maneuver [67–70].

As with prior studies, our group recommends limiting the use of the log-roll-maneuver to those situations where inspecting the back of a trauma patient may have immediate consequences for the treatment of the patient. This includes situations where a patient is found in a prone position and has to be rolled over onto a transportation device. Some studies suggest there is significantly more motion in the unstable spine by using the log-roll maneuver compared to alternative maneuvers like the straddle lift and slide, 6 + lift and slide or the scoop stretcher [67, 70, 71].

### Key issues for future investigations

As mentioned by previous authors, well-designed, prospective studies, including randomized controlled trials to elucidate the efficacy of spinal stabilisation and the preferred techniques are warranted [72]. However, ethical, consent and potential medico-legal and practical issues are recognized as barriers which may limit such studies in the prehospital settings.

Large, international cohort studies and / or comparative studies may also yield a better understanding of the various spinal.stabilisation measures and their potential harms and benefits.

## Conclusion

The evidence for spinal stabilisation of trauma patient is sparse. Based on a systematic review of the existing literature, grading of the strength of the evidence, clinical judgment and a consensus process, our Danish working group formulated the following recommendations for spinal stabilisation of adult trauma patients: a strong recommendation against the efforts of spinal stabilisation in case of patients with isolated penetrating injuries, a weak recommendation against the use of the rigid cervical collar as well as the hard backboard, and a weak recommendation for the use of a vacuum mattress in case of ABCDE-stable patients. Lastly, our working group suggests our algorithm should be adopted based on the clinical findings rather than the mechanisms of injury to guide clinical practice.

## Additional file

Additional file 1: Search strategy for “spinal stabilisation of adult trauma patients”. (DOCX 31 kb)

## Data Availability

We have submitted our detailed search vocabulary as supplementary material.

## References

[1] Bjørnshave Noe B, Mikkelsen E, Hansen R, Thygesen M, Hagen E (2015). Incidence of traumatic spinal cord injury in Denmark, 1990–2012: a hospital-based study. Spinal Cord.

[2] National Association of Emergency Medical Technicians (U.S.). Pre-Hospital Trauma Life Support Committee., American College of Surgeons. Committee on Trauma. Spinal Trauma. In: Emerton C, editor. PHTLS® Prehospital Trauma Life Support. 8th ed. Burlington, MA: Jones & Bartlett Learning; 2016. p. 289–314.

[3] Subcommittee ATLS, Merrick C (2018). American College of Surgeons’ committee on trauma, international ATLS working group. Spine and spinal cord trauma. Adv trauma life support tenth Ed.

[4] Hood N, Considine J (2015). Spinal immobilisaton in pre-hospital and emergency care: a systematic review of the literature. Australas Emerg Nurs J.

[5] Kwan II, Bunn FI, Roberts IG. Spinal immobilisation for trauma patients (Review). Cochrane Database Syst Rev [Internet]. 2001 [cited 2019 Jun 26]; Available from: https://www.cochranelibrary.com/cdsr/doi/10.1002/14651858.CD002803/epdf/full 10.1002/14651858.CD002803 PMC7003994 11406043

[6] Purvis TA, Carlin B, Driscoll P (2017). The definite risks and questionable benefits of liberal pre-hospital spinal immobilisation. Am J Emerg Med [Internet].

[7] Hauswald M, Ong G, Tandberg D, Omal Z, Mbbs ; Out-of-hospital Spinal Immobilization: Its Effect on Neurologic Injury. Acad Emerg Med MAR [Internet]. 1998 [cited 2019 Apr 13];5:214–9. Available from: https://www.wildmedcenter.com/uploads/5/9/8/2/5982510/hauswald_1998.pdf 10.1111/j.1553-2712.1998.tb02615.x 9523928

[8] Haut ER, Kalish BT, Efron DT, Haider AH, Stevens KA, Kieninger AN, et al. Spine Immobilization in Penetrating Trauma: More Harm Than Good? J Trauma Inj Infect Crit Care [Internet]. 2010 [cited 2019 Apr 13];68:115–21. Available from: https://pdfs.semanticscholar.org/5e88/0bc13d2e641cf58ade6ff07d288a0fbbd7d1.pdf?_ga=2.115322820.274011399.1538590597-783655649.1536783422 10.1097/TA.0b013e3181c9ee58 20065766

[9] Brooke Lerner E, Billittier A, Moscati RR (1998). The effects of neutral positioning with and without padding on spinal immobilization of healthy subjects. Prehospital Emerg Care..

[10] Connor D, Greaves I, Porter K, Bloch M. Prehospital spinal immobilisation: an initial consensus statement*. J Emerg Med [Internet]. 2013 [cited 2019 Apr 13];30:1067–9. Available from: https://emj.bmj.com/content/30/12/1067.long 10.1136/emermed-2013-203207 24232011

[11] Freauf M, Puckeridge N (2015). To board or not to board: an evidence review of prehospital spinal immobilization. J Emerg Med.

[12] Taub EC (2017). Cervical spine immobilization in athletes-to immobilize or not? A systematic review. Clin J Sport Med.

[13] Ham WHW, Schoonhoven L, Schuurmans MJ, Leenen LPH. Pressure ulcers, indentation marks and pain from cervical spine immobilization with extrication collars and headblocks: An observational study. J Care Inj [Internet]. 2016 [cited 2019 Apr 13];47:1924–31. Available from: 10.1016/j.injury.2016.03.032 10.1016/j.injury.2016.03.032 27158006

[14] Stiell I, Nesbitt L, Pickett W, Munkley D, Spaite D. The OPALS Major Trauma Study: impact of advanced life-support on survival and morbidity. CMAJ [Internet]. 2008 [cited 2019 Jun 18];178:1141–52. Available from: www.cmaj.ca/cgi/content/full/178/9/1141/DC1 10.1503/cmaj.071154 PMC2292763 18427089

[15] Hemmes B, Jeukens CRLPN, Al-Haidari A, Hofman PAM, Vd Linden ES, Brink PRG (2016). Effect of spineboard and headblocks on the image quality of head CT scans. Emerg Radiol.

[16] Stevens AC, Trammell TR, Billows GL, Ladd LM, Olinger ML. Emergency Radiology: Radiation exposure as a consequence of spinal immobilization and extrication. J Emerg Med [Internet]. 2015 [cited 2019 Apr 13];48:172–7. Available from: 10.1016/j.jemermed.2014.06.049 10.1016/j.jemermed.2014.06.049 25256410

[17] Stokkeland JP, Andersen E, Bjørndal MM, Mikalsen AM, Aslaksen S, Hyldmo PK (2017). Maintaining immobilisation devices on trauma patients during CT: a feasibility study. Scand J Trauma Resusc Emerg Med.

[18] Nemunaitis G, Joan Roach M, Samir Hefzy M, Mejia M (2016). Redesign of a spine board: proof of concept evaluation. AssistiveTechnology..

[19] Rao PJ, Phan K, Mobbs RJ, Wilson D, Ball J, Wales S (2016). Cervical spine immobilization in the elderly population. J Spine Surg.

[20] Schünemann H, Brożek J, Guyatt G, Oxman A. GRADE handbook for grading quality of evidence and strength of recommendations [internet]. GRADE Work Gr. 2013; Available from: http://www.gradeworkinggroup.org.

[21] Maschmann C, Jeppesen E, Rubin MA, Barfod C, National arbejdsgruppe for udarbejdelse af NKR for spinal stabilisering af voksne traumepatienter i Danmark. National Klinisk Retningslinje (NKR) for spinal stabilisering af voksne traumepatienter i Danmark (National Clinical Guideline for the Spinal Stabilisation of adult trauma patients in Denmark) [Internet]. Copenhagen: Danish Board of Health; 2018 [cited 2019 Apr 13]. p. 1–45. Available from: https://s3.amazonaws.com/files.magicapp.org/guideline/d7a7720b-c6b9-4bd2-af1d-eb6439f0e8e7/published_guideline_2840-2_1.pdf

[22] Kornhall DK, Jørgensen JJ, Brommeland T, Hyldmo PK, Asbjørnsen H, Dolven T (2017). The Norwegian guidelines for the prehospital management of adult trauma patients with potential spinal injury [internet].

[23] CASP Checklists - CASP - Critical Appraisal Skills Programme [Internet]. [cited 2019 Apr 13]. Available from: https://casp-uk.net/casp-tools-checklists/

[24] Brouwers MC, Kho ME, Browman GP, Burgers JS, Cluzeau F, Feder G (2010). AGREE II: advancing guideline development, reporting and evaluation in health care. CMAJ CMAJ.

[25] Brouwers MC, Hanna S. The AGREE II Instrument [Internet]. AGREE Next Steps Consort. 2017 [cited 2019 Mar 4]. Available from: www.agreetrust.org

[26] Danish Board of Health. Metodehåndbog for udarbejdelse af Nationale Kliniske Retningslinjer for puljeprojekter 2017–2020 (Handbook for the development of National Clinical Guidelines funded by the Danish Finance Act 2017–2020) [Internet]. 2017 [cited 2019 Mar 4]. Available from: https://www.sst.dk/da/nkr

[27] Høeg K, Lindkvist M. Spinal stabilisering – Nye kliniske retningslinjer (Spinal stabilisation - New Clinical Guidelines) – FOAMmedic [Internet]. FOAMmedic.org. 2018 [cited 2019 Apr 13]. Available from: https://foammedic.org/ep-15-spinal-stabilisering-nye-kliniske-retningslinjer/

[28] Lindkvist M. Video – Hvordan skal vi udføre spinalstabilisering efter den nye NKR? (How should we perform spinal stabilisation according to the new National Clinical Guidelines?) – FOAMmedic [Internet]. FOAMmedic.org. 2019 [cited 2019 Apr 9]. Available from: https://foammedic.org/video-hvordan-skal-vi-udfore-spinalstabilisering-efter-den-nye-nkr/

[29] Maschmann C, Rudolph M. Die Zervikalstütze für Traumapatienten - seit 2018 obsolet? (The rigid cervical collar for the trauma patient - obsolete since 2018?). Der Notarzt [Internet]. 2019;35:65. Available from: https://www.thieme-connect.de/products/ejournals/journal/10.1055/s-00000043

[30] Rubin MA. Copenhagen Critical Care Symposium - FOAMmedic talk: The new National Clinical Guidelines on spinal stabilisation of adult trauma patients [Internet]. 2019 [cited 2019 Jun 23]. Available from: https://youtu.be/WnrmbQCqPTg

[31] European EMS-congress 2019 Madrid [Internet]. 2019 [cited 2019 Jun 23]. Available from: https://emseurope.org/scientific-programme/

[32] Holla M, Hannink G, Eggen TGE, Daanen RA, Allard Ã, Hosman JF, et al. Restriction of Cervical Intervertebral Movement With Different Types of External Immobilizers A Cadaveric 3D Analysis Study. Spine (Phila Pa 1976). 2017;42:E1182–9. 10.1097/BRS.0000000000002107 28230622

[33] Kwan I, Bunn F (2005). Effects of prehospital spinal immobilization: a systematic review of randomized trials on healthy subjects. Prehosp Disaster Med.

[34] Cowley A, Hague A, Durge N. Cervical spine immobilization during extrication of the awake patient: a narrative review. Eur J Emerg Med [Internet]. 2017 [cited 2019 Apr 13];24:158–61. Available from: https://www.researchgate.net/publication/308841953_Cervical_spine_immobilization_during_extrication_of_the_awake_patient_a_narrative_review 10.1097/MEJ.0000000000000424 27748690

[35] Benger J, Blackham J. Why Do We Put Cervical Collars On Conscious Trauma Patients? Scand J Trauma Resusc Emerg Med [Internet]. 2009 [cited 2019 Apr 13];17. Available from: http://www.sjtrem.com/content/17/1/44 10.1186/1757-7241-17-44 PMC2751736 19765308

[36] Horodyski M, DiPaola CP, Conrad BP, Rechtine GR. Cervical collars are insufficient for immobilizing an unstable cervical spine injury. J Emerg Med [Internet]. 2011 [cited 2019 Apr 14];41:513–9. Available from: https://linkinghub.elsevier.com/retrieve/pii/S0736467911001715 10.1016/j.jemermed.2011.02.001 21397431

[37] Barati K, Arazpour M, Vameghi R, Abdoli A, Farmani F (2017). The effect of soft and rigid cervical collars on head and neck immobilization in healthy subjects. Asian Spine J.

[38] Ivancic PC. Do cervical collars and cervicothoracic orthoses effectively stabilize the injured cervical spine? A biomechanical investigation. Spine (Phila Pa 1976) [Internet]. 2013;38:E767–74. Available from: https://insights.ovid.com/crossref?an=00007632-201306010-00004 10.1097/BRS.0b013e318290fb0f 23486409

[39] Podolsky S, Baraff LJ, Simon RR, Hoffman JR, Larmon B, Ablon W (1983). Efficacy of cervical spine immobilization methods. J Trauma.

[40] McCabe JB, Nolan DJ (1986). Comparison of the effectiveness of different cervical immobilization collars. Ann Emerg Med.

[41] Pryce R, Mcdonald N. Prehospital Spinal Immobilization: Effect of Effort on Kinematics of Voluntary Head-neck Motion Assessed using Accelerometry. Prehosp Disaster Med [Internet]. 2016 [cited 2019 Apr 17];31:36–42. Available from: 10.1017/S1049023X1500552X. 10.1017/S1049023X1500552X 26674843

[42] Theodore N, Hadley MN, Aarabi B, Dhall SS, Gelb DE (2013). Hurlbert R John, et al. prehospital cervical spinal immobilization after trauma. Neurosurgery..

[43] Patel MB, Humble SS, Cullinane DC, Day MA, Jawa RS, Devin CJ (2015). Cervical spine collar clearance in the obtunded adult blunt trauma patient: a systematic review and practice management guideline from the eastern Association for the Surgery of trauma. J Trauma Acute Care Surg.

[44] Ala A (2016). Shams-Vahdati · S, Taghizadieh · a, Miri · S H, Kazemi · N, Hodjati · S R, et al. cervical collar effect on pulmonary volumes in patients with trauma. Eur J Trauma Emerg Surg.

[45] Ham W, Schoonhoven L, Marieke M(, Schuurmans ) J, Luke L(, Leenen PH. Pressure ulcer development in trauma patients with suspected spinal injury; the influence of risk factors present in the Emergency Department. Int Emerg Nurs [Internet]. 2017 [cited 2019 Apr 13];30:13–9. Available from: 10.1016/j.ienj.2016.05.005 10.1016/j.ienj.2016.05.005 27450044

[46] Maissan IM, Ketelaars R, Vlottes B, Hoeks SE, Den Hartog D, Stolker RJ. Increase in intracranial pressure by application of a rigid cervical collar: a pilot study in healthy volunteers. Eur J Emerg Med 2017;00:0–000. 10.1097/MEJ.0000000000000490 28727580

[47] Holla M. Value of a rigid collar in addition to head blocks: a proof of principle study. Emerg Med J. 2012;29:104 LP – 107. 10.1136/emj.2010.092973 21335583

[48] Prasarn ML, Hyldmo PK, Zdziarski LA, Loewy E, Dubose D, Horodyski M, et al. Comparison of the Vacuum Mattress versus the Spine Board Alone for Immobilization of the Cervical Spine Injured Patient A Biomechanical Cadaveric Study. Spine (Phila Pa 1976). 2017;42:E1398–402. 10.1097/BRS.0000000000002260 28591075

[49] Wampler DA, Pineda C, Polk J, Kidd E, Leboeuf D, Flores M, et al. The long spine board does not reduce lateral motion during transport-a randomized healthy volunteer crossover trial. Am J Emerg Med [Internet]. 2016 [cited 2019 Apr 17];34:717–21. Available from: 10.1016/j.ajem.2015.12.078 10.1016/j.ajem.2015.12.078 26827233

[50] Kreinest M, Scholz M, Trafford P (2017). on-scene treatment of spinal injuries in motor sports. Eur J Trauma Emerg Surg.

[51] Pernik MN, Seidel HH, Blalock RE, Burgess AR, Horodyski M, Rechtine GR, et al. Comparison of tissue-interface pressure in healthy subjects lying on two trauma splinting devices. Injury [Internet]. 2016 [cited 2019 Apr 17];47:1801–5. Available from: 10.1016/j.injury.2016.05.018 10.1016/j.injury.2016.05.018 27324323

[52] Holla M, Driessen M, Eggen TGE, Daanen RA, Hosman AJF, Verdonschot N (2017). A new Craniothoracic mattress for immobilization of the cervical spine in critical care patients. J Trauma Nurs.

[53] Domeier RM, Evans RW, Swor RA, Rivera-Rivera EJ, Frederiksen SM (1997). Prehospital clinical findings associated with spinal injury. Prehospital Emerg Care.

[54] Domeier RM, Evans RW, Swor RA, Hancock JB, Fales W, Krohmer J (1999). The reliability of prehospital clinical evaluation for potential spinal injury is not affected by the mechanism of injury. Prehospital Emerg Care..

[55] Hong R, Meenan M, Prince E, Murphy R, Tambussi C, Rohrbach R, et al. Comparison of three prehospital cervical spine protocols for missed injuries. West J Emerg Med [Internet]. California Chapter of the American Academy of Emergency Medicine (Cal/AAEM); 2014 [cited 2019 Apr 13];15:471–9. Available from: http://www.pubmedcentral.nih.gov/articlerender.fcgi?artid=PMC4100854 10.5811/westjem.2014.2.19244 PMC4100854 25035754

[56] Stroh G, Braude D. Can an out-of-hospital cervical spine clearance protocol identify all patients with injuries? An argument for selective immobilization. Ann Emerg Med [Internet]. Elsevier; 2001 [cited 2019 Apr 13];37:609–15. Available from: http://linkinghub.elsevier.com/retrieve/pii/S0196064401383270 10.1067/mem.2001.114409 11385329

[57] Burton JH, Dunn MG, Harmon NR, Hermanson TA, Bradshaw JR. A statewide, prehospital emergency medical service selective patient spine immobilization protocol. J Trauma. The Journal of Trauma: Injury, Infection, and Critical Care; 2006;61:161–7. 10.1097/01.ta.0000224214.72945.c4 16832265

[58] Michaleff ZA, Maher CG, Verhagen AP, Rebbeck T, Lin C-WC. Accuracy of the Canadian C-spine rule and NEXUS to screen for clinically important cervical spine injury in patients following blunt trauma: a systematic review. CMAJ [Internet]. Canadian Medical Association; 2012 [cited 2019 Apr 13];184:E867–76. Available from: http://www.pubmedcentral.nih.gov/articlerender.fcgi?artid=PMC3494329 10.1503/cmaj.120675 PMC3494329 23048086

[59] Hoffman JR, Mower WR, Wolfson AB, Todd KH, Zucker MI (2000). Validity of a set of clinical criteria to rule out injury to the cervical spine in patients with blunt trauma. N Engl J Med.

[60] Rogers L. No place for the rigid cervical collar in pre-hospital care. Int Paramed Pract [Internet]. 2017;7:12–5. Available from: http://www.magonlinelibrary.com/doi/10.12968/ippr.2017.7.1.12

[61] Martin MJ, Bush LD, Inaba K, Byerly S, Schreiber M, Peck KA (2017). Cervical spine evaluation and clearance in the intoxicated patient: a prospective Western trauma association multi-institutional trial and survey accreditation statement. J Trauma Acute Care Surg.

[62] Konstantinidis A, Plurad D, Barmparas G, Inaba K, Lam L, Bukur M, et al. The Presence of Nonthoracic Distracting Injuries Does Not Affect the Initial Clinical Examination of the Cervical Spine in Evaluable Blunt Trauma Patients: A Prospective Observational Study. J Trauma Inj Infect Crit Care [Internet]. 2011 [cited 2019 Apr 14];71:528–32. Available from: https://insights.ovid.com/crossref?an=00005373-201109000-00002 10.1097/TA.0b013e3181f8a8e0 21248650

[63] Dahlquist RT, Fischer PE, Rogers A, Christmas AB, Gibbs MA, Sing RF. Femur fractures should not be considered distracting injuries for cervical spine assessment. Am J Emerg Med [Internet]. 2015 [cited 2019 Apr 14];33:1750–4. Available from: 10.1016/j.ajem.2015.08.009 10.1016/j.ajem.2015.08.009 26346048

[64] Cason B, Rostas J, Simmons J, Frotan MA, Brevard SB, Gonzalez RP (2016). Thoracolumbar spine clearance: clinical examination for patients with distracting injuries. J Trauma Acute Care Surg.

[65] Nahm FS, Lee PB, Kim TH, Kim YC, Lee CJ. Comparative analysis of the independent medical examination reports and legal decisions in pain medicine. Korean J Pain [Internet]. Korean Pain Society; 2010 [cited 2019 Jun 25];23:28–34. Available from: http://www.ncbi.nlm.nih.gov/pubmed/20552070. 10.3344/kjp.2010.23.1.28 PMC2884209 20552070

[66] Chow J, He T. The General History and Physical Exam. In: Hall J, Piggott K, Vojvodic M, Zaslavsky K, editors. Essentials Clin Exam Handb 7 e [Internet]. 7th ed. New York: Thieme; 2013 [cited 2019 Jun 26]. p. 1–16. Available from: https://www.slideshare.net/libaanhassan/essentials-of-clinical-examination-handbook-7-e-2013pdfkoudiai-vrg

[67] Conrad BP, Del Rossi G, Horodyski MB, Prasarn ML, Alemi Y, Rechtine GR (2012). Eliminating log rolling as a spine trauma order. Surg Neurol Int.

[68] Hyldmo PK, Vist GE, Feyling AC, Rognås L, Magnusson V, Sandberg M, et al. Is the supine position associated with loss of airway patency in unconscious trauma patients? A systematic review and meta-analysis. Scand J Trauma Resusc Emerg Med. 2015. 10.1186/s13049-015-0116-0 PMC4486423 26129809

[69] Hyldmo PK, Horodyski MB, Conrad BP, Dubose DN, Røislien J, Prasarn M (2016). Safety of the lateral trauma position in cervical spine injuries: a cadaver model study. Acta Anaesthesiol Scand.

[70] Hyldmo K, Horodyski M, Conrad BP, Aslaksen S, Røislien J, Prasarn M, et al. Does the novel lateral trauma position cause more motion in an unstable cervical spine injury than the logroll maneuver?-NC-ND license. Am J Emerg Med. 2017;35:1630–5. [Internet]. (https://www.ajemjournal.com/article/S0735-6757(17)30366-2/pdf). [cited 2019 Apr 13]. 10.1016/j.ajem.2017.05.002 28511807

[71] Tveit MS, Singh E, Olaussen A, Liew S, Fitzgerald MC, Mitra B (2016). What is the purpose of log roll examination in the unconscious adult trauma patient during trauma reception?. Emerg Med J.

[72] Oteir O, Smith K, Stoelwinder JU, Middleton J, Jennings PA. Should suspected cervical spinal cord injury be immobilised?: A systematic review. Inj Int J Care Inj [Internet]. 2015 [cited 2019 Jun 23];46:528–35. Available from: 10.1016/j.injury.2014.12.032 10.1016/j.injury.2014.12.032 25624270

