# Supplementary material for: New clinical guidelines on the spinal stabilisation of adult trauma patients – consensus and evidence based
Source: Scand J Trauma Resusc Emerg Med. 2019 Aug 19;27:77. doi: 10.1186/s13049-019-0655-x (PMC6700785; doi:10.1186/s13049-019-0655-x)
Supplement: Supplementary file 1 — Search strategy for “spinal stabilisation of adult trauma patients”. (DOCX 31 kb) [file 13049_2019_655_MOESM1_ESM.docx]

# Search strategy for ”spinal stabilisation of adult trauma patients”

| **Project title** | Spinal stabilisation of adult trauma patients |
| --- | --- |
| **Contact (project group)** | Christian Maschmann: email: [c.maschmann@icloud.com](mailto:c.maschmann@icloud.com)  Mette Wenøe : email: [mette.wenoee@regionh.dk](mailto:mette.wenoee@regionh.dk) |
| **Contact (search specialist)** | Conni Skrubbeltrang: email: [cs@rn.dk](mailto:cs@rn.dk)  Chief librarian, Aalborg University Hospital, Denmark |
| **Latest update:** | 23^rd^ Oct. 2017 |

| **Background** | During the last decades it has been pivotal to spinally stabilise all trauma patients routinely with a rigid cervical collar and a spineboard in order to avoid the development of a secondary spinal cord injury.  However, this procedure has been questioned in recent years, since numerous studies discussing the efficacy of this procedure were published. On the contrary, the amount of studies showing potential harmful effects is growing.  Several studies discussed this issue and a Norwegian guideline on spinal stabilisation was published in 2017, whilst an updated national Danish consensus was still missing.  The aim of this literature search is to find evidence that can form the basis of new Danish guidelines for the spinal stabilisation of adult trauma patients. |
| --- | --- |
| **Search** | Since a newer Norwegian guideline from 2017 of good quality exists, we duplicated their search strategy. |
| **Inclusion- and exclusion criteria** | **Languages:** English, Danish, Norwegian, Swedish and German  **Timeframe:** January 2015 - October 2017  **Types of publication:** following types of publication were excluded:  Addresses or autobiography or bibliography or biography or classical article or clinical conference or comment or congresses or consensus development conference or consensus development conference, nih or "corrected and republished article" or dataset or dictionary or directory or duplicate publication or editorial or evaluation studies or festschrift or historical article or interactive tutorial or interview or lectures or legal cases or legislation or letter or news or newspaper article or patient education handout or periodical index or personal narratives or portraits or twin study or validation studies or video-audio media or webcasts) |

## Sources

| Database | Provider | Results | Date |
| --- | --- | --- | --- |
| Medline | Ovid | 4996 | 19.10.2017 |
| Embase | Ovid | 4298 | 19.10.2017 |
| Cochrane Trials | Wiley InterScience | 577 | 19.10.2017 |
| ClinicalTrials.Gov | Clinicaltrials.gov | 35 | 19.10.2017 |
| Total  After duplicate search in Endnote  After entering in Covidence |  | 9906  6512  **6484** |  |

**Ovid MEDLINE(R) Epub Ahead of Print, In-Process & Other Non-Indexed Citations, Ovid MEDLINE(R) Daily and Ovid MEDLINE(R) 1946 to Present 19.October 2017**

| 1 | exp Back Injuries/ | 23880 |
| --- | --- | --- |
| 2 | exp Multiple Trauma/ | 12799 |
| 3 | exp neck injuries/ | 7876 |
| 4 | exp Spinal Cord Injuries/ | 45870 |
| 5 | Spinal Cord Compression/ | 11196 |
| 6 | Trauma, Nervous System/ | 1273 |
| 7 | (((Spinal or spine or myelopath* or neck or cervix or cervical or brain or back or Whiplash or vertebra* or axis or dens or epistropheus or odontoid or atlant* or atlas or compressi*) adj2 (injur* or trauma* or fracture* or wound* or Polytrauma or transection* or laceration* or Post-Traumatic or "Post Traumatic" or contusion* or compressi* or dislocat* or displacement* or subluxation* or luxation*)) or (Conus adj Medullaris adj Syndrome*)).tw. | 255392 |
| 8 | or/1-7 | 292910 |
| 9 | exp Orthotic Devices/ | 12523 |
| 10 | Splints/ | 8627 |
| 11 | Stretchers/ | 100 |
| 12 | ((vacuum adj4 splint*) or Immobili* or precaution* or stabili* or stable or collar* or board* or backboard* or "back board*" or orthotic or orthos* or brace* or restraint* or "vacuum mattress*" or splint* or "sand bag*" or MILS or "manual in-line stabilization" or "manual in-line stabilisation" or tape* or taping or strap* or packag* or stretcher* or gurney* or "head block*" or headblock* or fixat* or extricat*).tw. | 1442343 |
| 13 | or/9-12 | 1450859 |
| 14 | 8 and 13 | 34368 |
| 15 | limit 14 to (yr="2015 -Current" and (danish or english or german or norwegian or swedish)) | 6071 |
| 16 | limit 15 to (addresses or autobiography or bibliography or biography or classical article or clinical conference or comment or congresses or consensus development conference or consensus development conference, nih or "corrected and republished article" or dataset or dictionary or directory or duplicate publication or editorial or evaluation studies or festschrift or historical article or interactive tutorial or interview or lectures or legal cases or legislation or letter or news or newspaper article or patient education handout or periodical index or personal narratives or portraits or twin study or validation studies or video-audio media or webcasts) | 204 |
| 17 | 15 not 16 | 5867 |
| 18 | exp animals/ | 22501594 |
| 19 | Humans/ | 17822099 |
| 20 | 18 not 19 | 4679495 |
| 21 | 17 not 20 | 5547 |
| 22 | remove duplicates from 21 | 4996 |

**Embase 1974 to 19. October 2017**

| 1 | exp spine injury/ | 40330 |
| --- | --- | --- |
| 2 | (((Spinal or spine or myelopath* or neck or cervix or cervical or brain or back or Whiplash or vertebra* or axis or dens or epistropheus or odontoid or atlant* or atlas or compressi*) adj2 (injur* or trauma* or fracture* or wound* or Polytrauma or transection* or laceration* or "Post Traumatic" or contusion* or compressi* or dislocat* or displacement* or subluxation* or luxation*)) or (Conus adj Medullaris adj Syndrome*)).tw. | 315339 |
| 3 | 1 or 2 | 331106 |
| 4 | exp immobilization/ | 65486 |
| 5 | exp cervical collar/ | 858 |
| 6 | spine board/ | 60 |
| 7 | spine stabilization/ | 7160 |
| 8 | exp splint/ | 8501 |
| 9 | stretcher/ | 319 |
| 10 | ((vacuum adj4 splint*) or Immobili* or precaution* or stabili* or stable or collar* or board* or backboard* or "back board*" or orthotic or orthos* or brace* or restraint* or "vacuum mattress*" or splint* or "sand bag*" or MILS or "manual in-line stabilization" or "manual in-line stabilisation" or tape* or taping or strap* or packag* or stretcher* or gurney* or "head block*" or headblock* or fixat* or extricat*).tw. | 1664903 |
| 11 | or/4-10 | 1687108 |
| 12 | 3 and 11 | 41572 |
| 13 | limit 12 to ((danish or english or german or norwegian or swedish) and yr="2015 -Current") | 8020 |
| 14 | limit 13 to (addresses or autobiography or bibliography or biography or clinical conference or comment or congresses or consensus development conference or consensus development conference, nih or "corrected and republished article" or dataset or dictionary or directory or duplicate publication or editorial or evaluation studies or festschrift or historical article or in vitro or interactive tutorial or interview or lectures or legal cases or legislation or letter or news or newspaper article or patient education handout or periodical index or portraits or twin study or validation studies or video-audio media or webcasts or book or book series or conference abstract or conference paper or conference proceeding or "conference review" or note or report or short survey or trade journal) [Limit not valid in Embase; records were retained] | 2592 |
| 15 | 13 not 14 | 5428 |
| 16 | 15 not ((exp animal/ or nonhuman/) not exp human/) | 4862 |
| 17 | remove duplicates from 16 | 4298 |

**Cochrane Library – Trials 19.October 2017**

| #1 | (Spinal or spine or myelopath* or neck or cervix or cervical or brain or back or Whiplash or vertebra* or axis or dens or epistropheus or odontoid or atlant* or atlas or compressi*) near/2 (injur* or trauma* or fracture* or wound* or Polytrauma or transection* or laceration* or Post-Traumatic or "Post Traumatic" or contusion* or compressi* or dislocat* or displacement* or subluxation* or luxation*) or (Conus next Medullaris next Syndrome*):ti,ab,kw (Word variations have been searched) | 15403 |
| --- | --- | --- |
| #2 | MeSH descriptor: [Back Injuries] explode all trees | 889 |
| #3 | MeSH descriptor: [Spinal Injuries] explode all trees | 835 |
| #4 | MeSH descriptor: [Spinal Fractures] explode all trees | 742 |
| #5 | MeSH descriptor: [Multiple Trauma] explode all trees | 214 |
| #6 | MeSH descriptor: [Neck Injuries] explode all trees | 239 |
| #7 | MeSH descriptor: [Whiplash Injuries] explode all trees | 202 |
| #8 | MeSH descriptor: [Spinal Cord Injuries] explode all trees | 1115 |
| #9 | MeSH descriptor: [Central Cord Syndrome] explode all trees | 0 |
| #10 | MeSH descriptor: [Spinal Cord Compression] explode all trees | 116 |
| #11 | MeSH descriptor: [Trauma, Nervous System] this term only | 24 |
| #12 | MeSH descriptor: [Spinal Cord Compression] explode all trees | 116 |
| #13 | #2 or #3 or #4 or #5 or #6 or #7 or #8 or #9 or #10 or #11 or #12 | 2451 |
| #14 | #1 or #13 | 15615 |
| #15 | (Immobili* or precaution* or stabili* or stable or collar* or board* or backboard* or "back board*" or back-board* or extrication or hypokine* or orthotic or orthos* or brace* or restraint* or "vacuum mattress*" or splint* or "sand bag*" or MILS or " manual in-line stabilization" or "manual in-line stabilisation" or tape* or taping or strap* or packag* or stretcher* or litter* or trolley* or gurney* or BNDR or fixation):ti,ab,kw (Word variations have been searched) | 69599 |
| #16 | MeSH descriptor: [Immobilization] explode all trees | 743 |
| #17 | MeSH descriptor: [Restraint, Physical] explode all trees | 261 |
| #18 | MeSH descriptor: [Braces] explode all trees | 405 |
| #19 | MeSH descriptor: [Splints] explode all trees | 428 |
| #20 | MeSH descriptor: [Stretchers] explode all trees | 6 |
| #21 | (Stif-neck or stiff-neck or "Stiff Neck" or x-collar or scoop or Kendrick or combicarrier* or skidboard or LBB or SAM or perfit or ACE og Redi-ACE*):ti,ab,kw (Word variations have been searched) | 298 |
| #22 | #15 or #16 or #17 or #18 or #19 or #20 or #21 | 69870 |
| #23 | #14 and #22 Publication Year from 2015 to 2017, in Trials | 577 |

**Clinicaltrials.gov**

23. October 2017

Search strategy:

immobilisation OR immobilization OR mobilization OR mobilisation OR mattress OR board OR brace OR collar OR backboard | Spinal Cord Injuries OR head injuries OR back injuries OR multiple trauma OR neck injuries | Adult, Senior | First posted from 01/01/2015 to 10/23/2017

35 entries.
